# Bioinformatics and network-based screening and discovery of potential molecular targets and small molecular drugs for breast cancer

**DOI:** 10.3389/fphar.2022.942126

**Published:** 2022-09-20

**Authors:** Md Shahin Alam, Adiba Sultana, Hongyang Sun, Jin Wu, Fanfan Guo, Qing Li, Haigang Ren, Zongbing Hao, Yi Zhang, Guanghui Wang

**Affiliations:** ^1^ Laboratory of Molecular Neuropathology, Department of Pharmacology, Jiangsu Key Laboratory of Neuropsychiatric Diseases and College of Pharmaceutical Sciences, Soochow University, Suzhou, Jiangsu, China; ^2^ Department of Pharmacology, College of Pharmaceutical Science, Soochow University, Suzhou, Jiangsu, China; ^3^ Department of Gastroenterology, the First People’s Hospital of Taicang, Taicang Affiliated Hospital of Soochow University, Suzhou, Jiangsu, China

**Keywords:** Breast cancer, gene expression profiles, molecular targets, bioinformatics and network-based discovery, molecular docking analysis, drug repurposing, apoptotic cell death

## Abstract

Accurate identification of molecular targets of disease plays an important role in diagnosis, prognosis, and therapies. Breast cancer (BC) is one of the most common malignant cancers in women worldwide. Thus, the objective of this study was to accurately identify a set of molecular targets and small molecular drugs that might be effective for BC diagnosis, prognosis, and therapies, by using existing bioinformatics and network-based approaches. Nine gene expression profiles (GSE54002, GSE29431, GSE124646, GSE42568, GSE45827, GSE10810, GSE65216, GSE36295, and GSE109169) collected from the Gene Expression Omnibus (GEO) database were used for bioinformatics analysis in this study. Two packages, LIMMA and clusterProfiler, in *R* were used to identify overlapping differential expressed genes (oDEGs) and significant GO and KEGG enrichment terms. We constructed a PPI (protein–protein interaction) network through the STRING database and identified eight key genes (KGs) EGFR, FN1, EZH2, MET, CDK1, AURKA, TOP2A, and BIRC5 by using six topological measures, betweenness, closeness, eccentricity, degree, MCC, and MNC, in the Analyze Network tool in Cytoscape. Three online databases GSCALite, Network Analyst, and GEPIA were used to analyze drug enrichment, regulatory interaction networks, and gene expression levels of KGs. We checked the prognostic power of KGs through the prediction model using the popular machine learning algorithm support vector machine (SVM). We suggested four TFs (TP63, MYC, SOX2, and KDM5B) and four miRNAs (hsa-mir-16-5p, hsa-mir-34a-5p, hsa-mir-1-3p, and hsa-mir-23b-3p) as key transcriptional and posttranscriptional regulators of KGs. Finally, we proposed 16 candidate repurposing drugs YM201636, masitinib, SB590885, GSK1070916, GSK2126458, ZSTK474, dasatinib, fedratinib, dabrafenib, methotrexate, trametinib, tubastatin A, BIX02189, CP466722, afatinib, and belinostat for BC through molecular docking analysis. Using BC cell lines, we validated that masitinib inhibits the mTOR signaling pathway and induces apoptotic cell death. Therefore, the proposed results might play an effective role in the treatment of BC patients.

## Introduction

Breast cancer (BC) is the most common cancer diagnosis and the leading cause of cancer-related deaths in women worldwide. It was calculated that 2,261,419 (11.7% of all cancers) new cases and 684,996 (6.9% of all cancers) deaths occurred in BC in 2020 (Sung et al., 2021). In China, 416,371 new cases and 117,414 BC-related deaths occurred in 2020, accounting for 18.4% of diagnoses and 17.1% of deaths (Cao et al., 2021). It was also estimated that there were 1,700,000 new cases and 521,900 deaths in 2012 BC worldwide (Torre et al., 2015). We noticed that BC has a high prevalence and mortality rate and is steadily increasing every year, although it is claimed that there have been significant advances in systematic treatment over the decades. The 5-year overall survival rate for BC patients is still low, although it depends on various factors (Sarveazad et al., 2018). Therefore, the progress of the existing treatment has not yet reached a satisfactory level. Therefore, it is urgent to improve research to discover potential molecular targets and effective candidate drugs for the development of innovative therapies for BC.

Novel drug discovery is challenging, time consuming, and expensive due to the very low rate of approval through clinical trials. Discovering a new drug using the *de novo* technique takes approximately 14 years and costs 800 million dollars, yet many pharmaceutical companies are working on it (Song et al., 2009; Lavecchia and Di Giovanni, 2013). Drug repurposing (DR) is a promising strategy to identify new indications for a specific disease by using approved drugs (existing drugs) (Simsek et al., 2018). The DR strategy is safer, cheaper, and less time consuming than the *de novo* strategy due to the knowledge of *in vivo* screening, chemical optimization, and toxicology of existing drugs (Ko, 2020). Thus, the DR technique in computers widely used by pharmaceutical companies and researchers over the past few decades has achieved significant success (Shi et al., 2020; Alam et al., 2022b). Molecular docking analysis is a momentous strategy for validating drug-target structural binding performance in computational DR processes. Bioinformatics analysis plays a significant role in accurately identifying the key genes (KGs)/targets with candidate drugs of the disease to inform diagnosis, prognosis, and therapies. The gene expression profile analysis is one of the most popular platforms for disease-guided KG identification. Thus, we used multiple datasets collected from different environments to identify more common and stable BC-guided KG through gene expression profiles (Alam et al., 2022a).

In the present study, we identified eight stable BC-guided KGs (EGFR, FN1, EZH2, MET, CDK1, AURKA, TOP2A, and BIRC5) and highlighted their role as molecular targets. We also proposed 16 KG-guided candidate drugs (YM201636, masitinib, SB590885, GSK1070916, GSK2126458, ZSTK474, dasatinib, fedratinib, dabrafenib, methotrexate, trametinib, tubastatin A, BIX02189, CP466722, Afatinib, and Belinostat) for BC treatment and validated them *in silico* through molecular docking analysis. Using BC cell lines, we found that masitinib inhibits the mTOR signaling pathway and induces apoptotic cell death.

## Materials and methods

### Microarray data

The gene expression profiles analyzed in our study were downloaded from the Gene Expression Omnibus (GEO) database (https://www.ncbi.nlm.nih.gov/geo/) (Edgar and Barrett, 2006). The workflow of the present study is presented in Supplementary Figure S1. Eight sets of gene expression profiles with accession numbers GSE54002 (Tan et al., 2014), GSE29431 (Wang et al., 2021), GSE124646 (Sinn et al., 2019), GSE42568 (Clarke et al., 2013), GSE45827 (Gruosso et al., 2016), GSE10810 (Pedraza et al., 2010), GSE65216 (Maubant et al., 2015), and GSE36295 (Karim et al., 2016) were used to identify differentially expressed genes (DEGs) between BC and normal samples. A total of 864 BC samples and 123 normal samples were included in these eight sets of profiles. An independent gene expression profile (GSE109169) (Yu et al., 2020) including 25 BC and 25 normal samples was collected to investigate the prognostic performance of KGs through the cancer prediction model. Furthermore, the six profiles (GSE54002, GSE29431, GSE42568, GSE45827, GSE10810, and GSE65216) based on the GPL570 platform, profile GSE124646 based on the GPL96 platform, profile GSE36295 based on the GPL6244 platform, and profile GSE109169 based on the GPL96 platform were identified. Details of the datasets are described in Table 1.

**TABLE 1:** Description of eight sets of gene expression profiles for BC analyzed in this study.

ACT	Sample size (tumor/normal)	Platform	Locations	References
GSE54002	433 (417/16)	GPL570 Affymetrix Human Genome U133 Plus 2.0 Array	Singapore	Tan et al. (2014)
GSE29431	66 (54/12)		Spain	Wang et al. (2021)
GSE124646	40 (20/20)	GPL96 Affymetrix Human Genome U133A Array	United States	Sinn et al. (2019)
GSE42568	121 (104/17)	GPL570 Affymetrix Human Genome U133 Plus 2.0 Array	Ireland	Clarke et al. (2013)
GSE45827	41 (30/11)		France	Gruosso et al. (2016)
GSE10810	58 (27/31)		Spain	Pedraza et al. (2010)
GSE65216	178 (167/11)		France	Maubant et al. (2015)
GSE36295	50 (45/5)	GPL6244 Affymetrix Human Gene 1.0 ST Array	Saudi Arabia	Karim et al. (2016)
GSE109169	50 (25/25)	GPL5175 Affymetrix Human Exon 1.0 ST Array	Taiwan	Yu et al. (2020)

### Data processing and differentially expressed gene identification

We normalized all datasets by using log2-transformation, after which we used the normalized data for further analysis. Then, the Limma (version: 3.14) package (Ritchie et al., 2015) in RStudio (version: 1.2.5019) was used to identify DEGs between BC samples and normal samples for eight datasets individually. The moderated T-statistics and the Benjamini and Hochberg false discovery rate method were used to calculate the *p* value and adjusted *p* value (adj.P.Val) and logFC (fold change), accordingly. The cutoff criteria adj.P.Val < 0.01 and | logFC | > 1.5 were considered to select significant DEGs. Overlapping upregulated and downregulated DEGs were screened using the Venn diagram web tool (https://bioinformatics.psb.ugent.be/webtools/Venn/).

### Protein–protein interaction network analysis of overlapping differentially expressed genes to identify key genes

The PPI network of oDEGs was constructed using the online database STRING (Search Tool for the Retrieval of Interacting Genes) (v 11.5) and was visualized through the Cytoscape software (Shannon et al., 2003; Szklarczyk et al., 2019). Then, we screened the top-ranked common KGs by using the six topological measures betweenness, closeness, eccentricity, degree, maximal clique centrality (MCC), and maximum neighborhood component (MNC) through the Analyze Network tool in the Cytoscape.

### Functional and pathway enrichment analysis of differentially expressed genes

GO (Gene Ontology) function in three categories BPs (Biological Processes), CC (Cellular Component), and MF (MF) and KEGG (Kyoto Encyclopedia of Genes and Genomes) pathway enrichment analyzed through the R/Bioconductor package “clusterProfiler” (version: 3.14). *p*-value cutoff = 0.05 and *q-*value cutoff = 0.05 were selected as the cutoff criteria (Yu et al., 2012).

### Regulatory interaction network analysis

The regulatory interaction network TFs (Transcription factors)-KGs-miRNAs (microRNAs) to detect transcriptional and posttranscriptional regulatory factors of KGs. The Network Analyst (Zhou et al., 2019) web-based tool was used to construct the regulatory interaction networks and was visualized by using the Cytoscape software (Shannon et al., 2003).

### Validation of prognostic power and expression pattern of key genes

We validated the expression pattern of KGs through TCGA (The Cancer Genome Atlas) RNA-seq data (independent data) by using the GEPIA database (Tang et al., 2017). Then, we performed the cancer prediction/classification model using the popular machine learning algorithm SVM to test the prognostic power of our proposed KGs. We used the expression profiles of KGs from eight sets of data (GSE54002, GSE29431, GSE124646, GSE42568, GSE45827, GSE10810, GSE65216, and GSE36295) as the prediction set and another independent validation set GSE26964. The ROCR package in *R* was used to generate the ROC curves (Sing et al., 2005).

### Drug repurposing by molecular docking analysis

To explore KG-guided candidate drugs for BC by molecular docking analysis, we considered eight KGs as drug target proteins (receptors). Subsequently, the 77 KG-associated meta-drug agents (ligands) were selected from the GSCALite database on the basis of a positive Spearman rank correlation with at least one KG (Liu et al., 2018). We downloaded 3-D structures of target proteins and drug agents for molecular docking analysis. The 3-D structures of the eight KGs EGFR, FN1, EZH2, MET, CDK1, AURKA, TOP2A, and BIRC5 were downloaded from the Protein Data Bank (PDB) database with the PDB IDs 3g5z, 2haz, 4mi0, 6hyg, 6gu6, 3dj5, 1zxm, and 1xox, respectively (Berman et al., 2002). The 3D structures of the meta-drug agents were downloaded from the PubChem database (Kim et al., 2019). The 3-D structure of receptors and ligands was preprocessed for molecular docking analysis using the PyMol software. Molecular docking analysis was performed using the AutoDock Vina software in PyRx to check the structural binding performance between receptors and ligands and computed the binding affinity scores (BAS) (kcal/mol) (Trott and Olson, 2010; Dallakyan and Olson, 2015). The “plot.matrix” package in *R* was used to visualize the molecular docking results.

### Statistical analysis

All gene expression profiles were statistically analyzed by the *R* software (Version 4.0.4). Moderated t-statistics were utilized to testH_0_ (equally expressed gene (EEG) in both the case and control groups) versus H_1_ (differentially expressed gene (DEG) between the case and control groups). Statistically significant differences were considered when *p* < 0.05.

### Cell culture and drug treatment

The BC cell lines MCF-7 and MDA-MB-231 cells were cultured in Dulbecco’s modified Eagle’s medium (Gibco, Los Angeles, CA) containing 10% fetal bovine serum (FBS, Gibco) supplemented with penicillin (100  µ/ml) and streptomycin (100 μg/ml, Gibco). Masitinib was purchased from MedChemExpress (Monmouth Junction, NJ, United States) and dissolved in dimethyl sulfoxide (DMSO). The cells were treated with different doses of masitinib for 24 or 48 h. After treatment, the cells were harvested with lysis buffer for immunoblot analyses or fixed with 4% paraformaldehyde for immunofluorescent staining.

### Cell viability assays

The cells were seeded in 96-well plates and cultured overnight. After treatment with masitinib at the indicated dose, a 10 µl solution of cell counting kit-8 (CCK-8) reagent was added to each well and incubated for 1 h in the dark. The absorbance was measured at 450 nm to calculate cell viability.

### Propidium iodide staining assay

The cells were treated with masitinib at different concentrations. After treatment, the cells were incubated with Hoechst 33342 (Sigma, St. Louis, MO, United States) and PI (Sigma, St. Luis, MO, United States ) for 10 min. The cells were then imaged with an inverted IX71 microscope system (Olympus, Tokyo, Japan).

### Immunoblot analyses

Immunoblot analyses were performed as described previously (Zhang et al., 2022). In brief, the cells were lysed in 1 × SDS lysis buffer supplemented with a protease inhibitor cocktail (Roche, Basel, Switzerland). The cell lysates were subjected to SDS–PAGE and transferred onto a PVDF membrane (Millipore, Billerica, MA, United States). The membranes were incubated with the following primary antibodies: anti-β-actin, anti-cleaved caspase-3, anti-cyclin D, anti-mTOR, anti-PARP, and anti-phospho-mTOR. The secondary antibodies, sheep anti-rabbit or anti-mouse IgG-HRP, were obtained from Thermo Fisher (Waltham, MA, United States). The proteins were visualized using an ECL detection kit (Thermo Fisher, Waltham, MA, United States).

## Results

### Identification of differentially expressed genes

**FIGURE 1:**
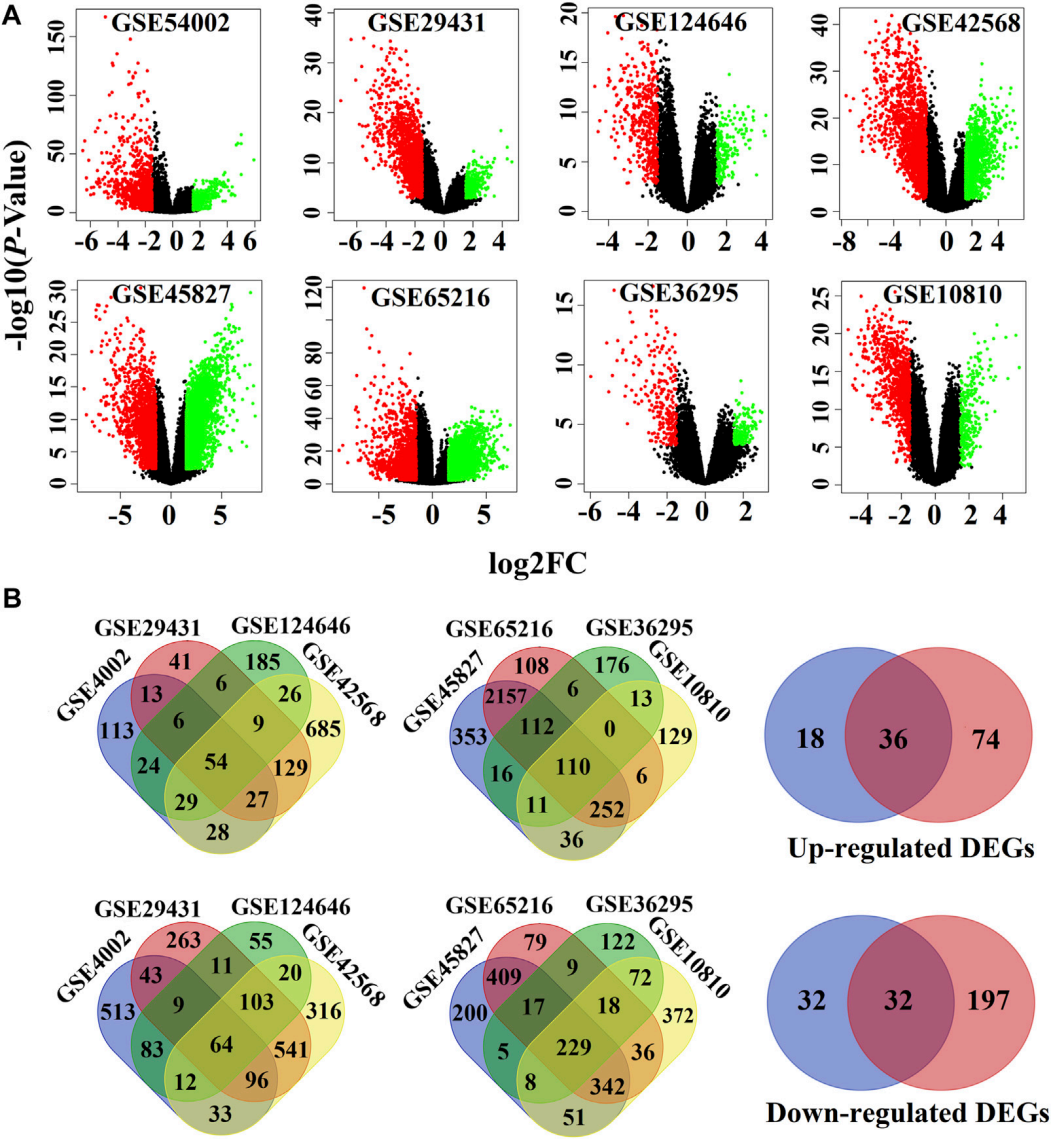
Screen of the overlapping DEGs (oDEGs) among eight sets of gene expression profiles. **(A)** Volcano plots of DEGs, where red dots indicate downregulated DEGs and green dots indicate upregulated DEGs. **(B)** Venn diagrams were used to screen overlapping upregulated and downregulated DEGs.

We used the LIMMA statistical approach to identify DEGs and considered the threshold adj.P.Val < 0.01 and |log2 fold change (FC) | >1.5 for selecting significantly upregulated and downregulated DEGs. The DEGs in each dataset are presented using volcano plots (Figure 1A), where red dots indicate downregulated DEGs and green dots indicate upregulated DEGs. Then, we generated a Venn diagram of the overlapping upregulated and downregulated DEGs in (Figure 1B). Finally, we obtained a total of 68 oDEGs (36 upregulated and 32 downregulated oDEGs) in Table 2, which are referred to as DEGs in this study.

**TABLE 2:** List of up- and downregulated oDEGs.

Upregulated oDEGs	Downregulated oDEGs
CDK1, BGN, PBK, CXCL10, GINS1, MELK, COL11A1, WISP1, NEK2, KIF2C, BIRC5, SQLE, RRM2, ZWINT, CCNB1, AURKA, SULF1, TOP2A, CENPF, KIAA0101, GPRC5A, NUSAP1, UBE2C, CKS2, COMP, FN1, ASPM, BUB1B, MAD2L1, HIST1H2BD, EZH2, CCNB2, ECT2, COL10A1, MMP11, and PRC1	FMO2, ABCA8, ITIH5, CHL1, IL33, TGFBR3, PDGFD, MET, LMOD1, ZBTB16, CDO1, DMD, SDPR, SORBS1, GHR, CXCL2, EGFR, LIFR, MAOA, S100B, CRYAB, PROS1, TF, MME, CAV1, SFRP1, PDK4, MT1M, HLF, GULP1, SPRY2, and ITM2A

### Protein–protein interaction network of overlapping differentially expressed genes to identify key genes

**FIGURE 2:**
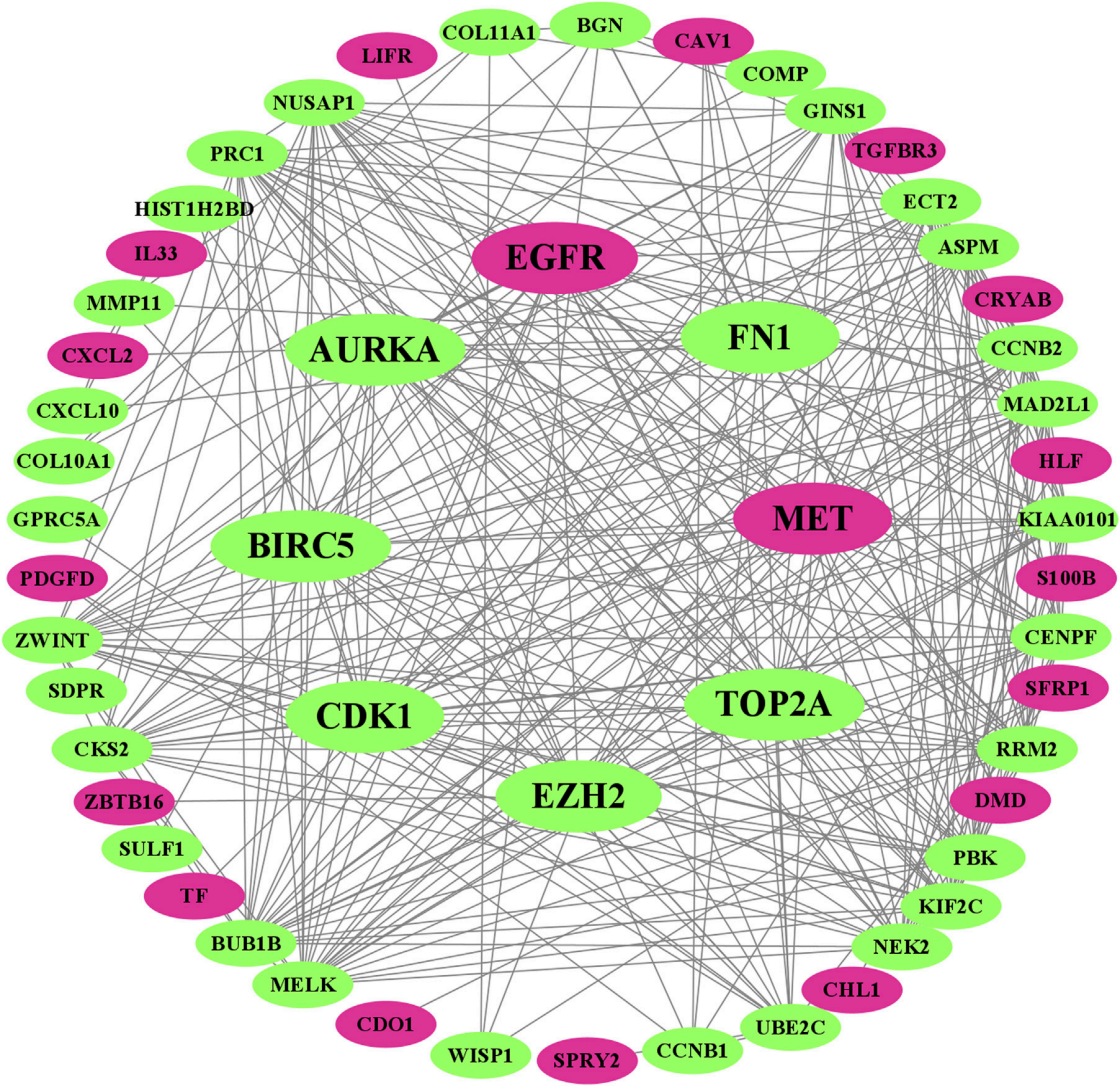
Visualized PPI network of oDEGs, where green indicates upregulated oDEGs, pink indicates downregulated oDEGs, and large size indicates KGs.

We visualized the PPI network of oDEGs in Figure 2, where green indicates upregulated oDEGs, pink indicates downregulated oDEGs, and large size indicates KGs. After that, we used six network scoring measures, including betweenness, closeness, eccentricity, degree, MCC, and MNC in the Analyze Network tool in the Cytoscape, and selected the top 25 genes for each measure. We extracted eight common genes (EGFR, FN1, EZH2, MET, CDK1, AURKA, TOP2A, and BIRC5) among the six lists and considered them as KGs in this study (Table 3). Further analyzed-based information of the KGs is presented in Table 4.

**TABLE 3:** Eight KGs were selected by taking the common of top 25 ranked genes for six network scoring measures through the PPI network.

Betweenness		Closeness		Eccentricity		Degree		MCC		MNC		Common genes (KGs)
GS	SC	GS	SC	GS	SC	GS	SC	GS	SC	GS	SC	
EGFR	1,256.7	EZH2	36.5	FN1	0.32	CDK1	25	PBK	5.36E + 19	CCNB1	25	
FN1	951.9	CDK1	35.9	CAV1	0.32	CCNB1	25	ASPM	5.36E + 19	CDK1	25	
EZH2	521.4	CCNB1	35.9	MET	0.32	EZH2	25	AURKA	5.36E + 19	AURKA	24	
MET	223.8	AURKA	35.4	EGFR	0.32	AURKA	24	KIF2C	5.36E + 19	BIRC5	24	
SFRP1	174.5	TOP2A	35.4	MMP11	0.32	TOP2A	24	PRC1	5.36E + 19	EZH2	24	
CAV1	173.3	BIRC5	35.4	TF	0.24	BIRC5	24	TOP2A	5.36E + 19	TOP2A	24	
BGN	153.7	EGFR	34	AURKA	0.24	UBE2C	24	NUSAP1	5.36E + 19	BUB1B	23	
CDK1	153.3	UBE2C	33.2	DMD	0.24	PBK	23	CDK1	5.36E + 19	CCNB2	23	EGFR
CCNB1	153.4	PBK	32.7	COL11A1	0.24	ASPM	23	CCNB1	5.36E + 19	KIF2C	23	FN1
AURKA	126	ASPM	32.7	CHL1	0.24	KIF2C	23	BUB1B	5.36E + 19	MELK	23	EZH2
TOP2A	126	KIF2C	32.7	S100B	0.24	PRC1	23	CCNB2	5.36E + 19	PBK	23	MET
BIRC5	126	PRC1	32.7	TOP2A	0.24	NUSAP1	23	MAD2L1	5.36E + 19	PRC1	23	CDK1
DMD	109	NUSAP1	32.7	SDPR	0.24	BUB1B	23	MELK	5.36E + 19	NUSAP1	23	AURKA
WISP1	54.5	BUB1B	32.7	GPRC5A	0.24	CCNB2	23	KIAA0101	5.36E + 19	MAD2L1	23	TOP2A
COL11A1	47.9	CCNB2	32.7	CDK1	0.24	MAD2L1	23	BIRC5	5.36E + 19	ASPM	23	BIRC5
UBE2C	44.1	MAD2L1	32.7	LIFR	0.24	MELK	23	UBE2C	5.36E + 19	RRM2	23	
MMP11	41.5	MELK	32.7	HLF	0.24	KIAA0101	23	RRM2	5.36E + 19	CENPF	23	
CHL1	30	KIAA0101	32.7	PDGFD	0.24	RRM2	23	CENPF	5.36E + 19	ZWINT	23	
COMP	22	RRM2	32.7	MELK	0.24	CENPF	23	ZWINT	5.36E + 19	MET	23	
S100B	12.5	CENPF	32.7	SPRY2	0.24	ZWINT	23	ECT2	5.35E + 19	UBE2C	23	
GPRC5A	5.6	ZWINT	32.7	BIRC5	0.24	MET	22	EZH2	5.11E + 19	ECT2	22	
PBK	2.1	ECT2	32.2	SFRP1	0.24	CKS2	21	NEK2	5.11E + 19	EGFR	21	
ASPM	2.1	CKS2	30.9	CXCL10	0.24	EGFR	21	MET	2.43E + 18	FN1	21	
KIF2C	2.1	MET	30.9	IL33	0.24	GINS1	19	FN1	1.22E + 17	GINS1	19	
PRC1	2.1	FN1	30.5	EZH2	0.24	FN1	16	EGFR	738	CKS2	12	

**TABLE 4:** List of KGS included with *p* values and logFC values on eight sets of data.

		GSE54002	GSE29431	GSE124646	GSE42568	GSE45827	GSE10810	GSE65216	GSE36295
EGFR	LF	−2.8	−3.4	−2.3	−2.4	−1.8	−1.9	−1.7	−1.8
	PV	1.90E-15	3.60E-17	2.00E-10	4.50E-09	0.0043	4.40E-17	0.009	0.0093
FN1	LF	5	1.6	1.6	2.2	3.6	1.6	1.7	1.9
	PV	3.30E-67	1.00E-07	2.10E-06	5.10E-10	8.70E-18	7.80E-11	1.90E-17	0.009
EZH2	LF	1.8	1.8	2.3	3.8	5	1.8	4.6	1.8
	PV	2.70E-16	4.40E-06	1.40E-06	1.00E-21	4.40E-23	0.0085	8.40E-22	1.40E-06
MET	LF	−3.1	−1.8	−1.6	−1.9	−1.9	−1.8	−1.6	−1.6
	PV	6.90E-12	5.40E-06	0.0059	2.60E-24	1.40E-17	1.40E-12	2.60E-38	8.60E-12
CDK1	LF	3.2	1.7	1.7	2.8	5.8	2.6	4.6	1.8
	PV	3.60E-27	6.50E-07	2.40E-09	2.40E-13	2.20E-22	2.50E-17	1.60E-20	0.0001
AURKA	LF	2.9	1.8	1.9	3.7	4.7	2.2	4.5	1.6
	PV	1.60E-23	5.90E-07	3.30E-07	9.50E-19	1.90E-20	2.90E-14	1.30E-14	0.0013
TOP2A	LF	4.1	2.7	3.4	4.6	6.7	3.2	6.7	2.7
	PV	2.20E-28	2.50E-07	6.70E-10	8.10E-19	1.00E-17	5.90E-16	4.60E-23	7.60E-06
BIRC5	LF	2.9	2.2	2.9	2.3	5.4	2.3	5.1	1.8
	PV	3.40E-19	4.30E-06	3.70E-08	2.00E-08	3.70E-15	4.60E-12	7.30E-12	0.0098

### Functional and pathway enrichment analysis of overlapping differentially expressed genes

**FIGURE 3:**
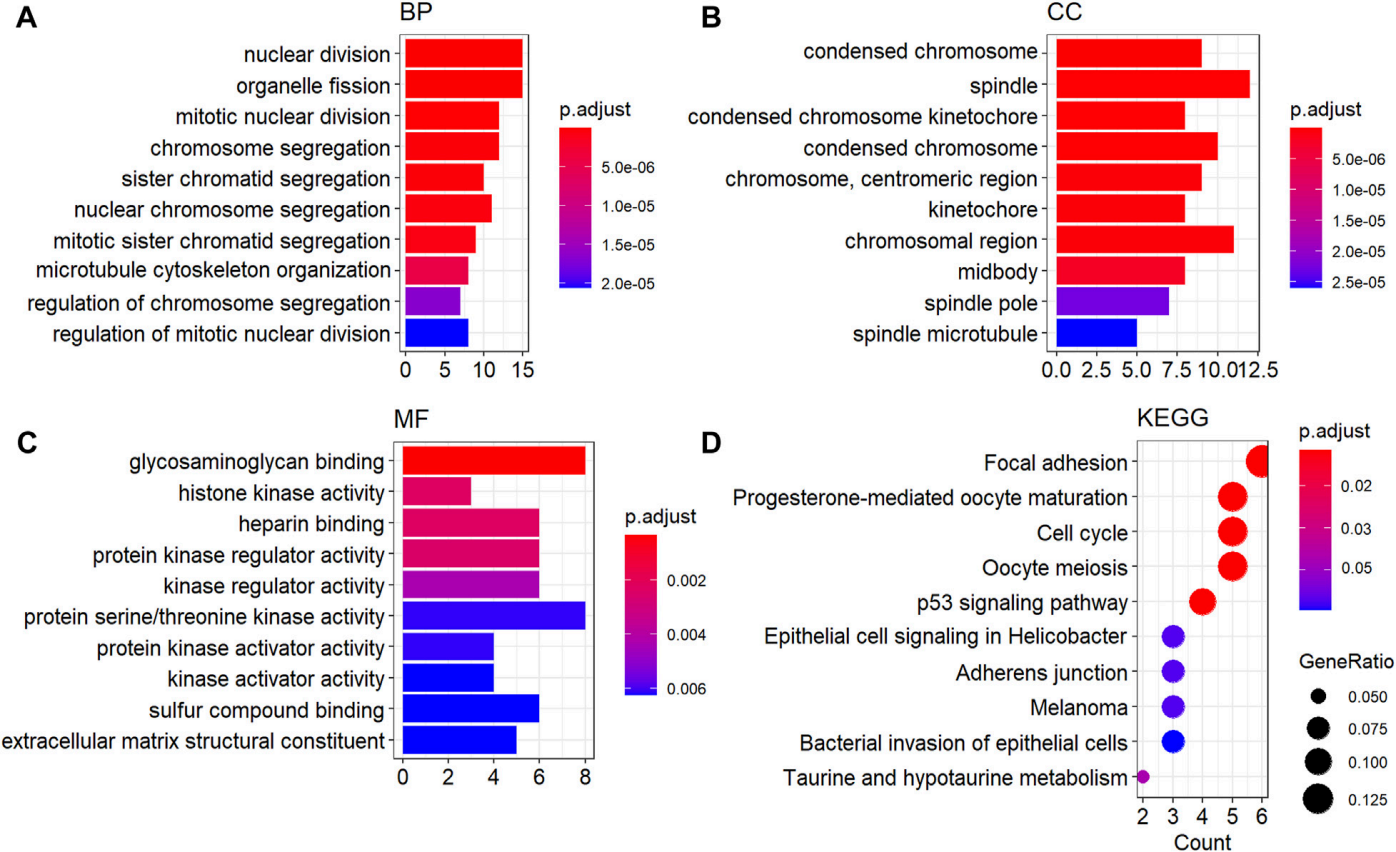
GO functional and KEGG enrichment analysis of oDEGs. **(A)** Top ten GO-BP terms, **(B)** top ten GO-CC terms, **(C)** top ten GO-MF terms, and **(D)** top ten KEGG pathway terms.

The GO functional and KEGG pathway enrichment analysis showed that 148 GO-BP terms, 27 GO-CC terms, 12 GO-MF terms, and 13 KEGG terms were enriched by oDEGs, and the top 10 terms of each category are represented in Figures 3A–C. Among them four significantly enriched BP terms, six CC terms, three MF terms, and five KEGG terms were directly associated with at least two KGs. The four BP terms were nuclear division, organelle fission, mitotic nuclear division, and chromosome segregation. The six CC terms were spindle, condensed chromosome, “chromosome, centromeric region”, chromosomal region, midbody, and spindle microtubule. The three MF terms were histone kinase activity, heparin binding, and protein serine/threonine kinase activity. Finally, the five KEGG terms were identified as progesterone-mediated oocyte maturation, focal adhesion, oocyte meiosis, adherens junction, and bacterial invasion of epithelial cells.

### Regulatory interaction network analysis

**FIGURE 4:**
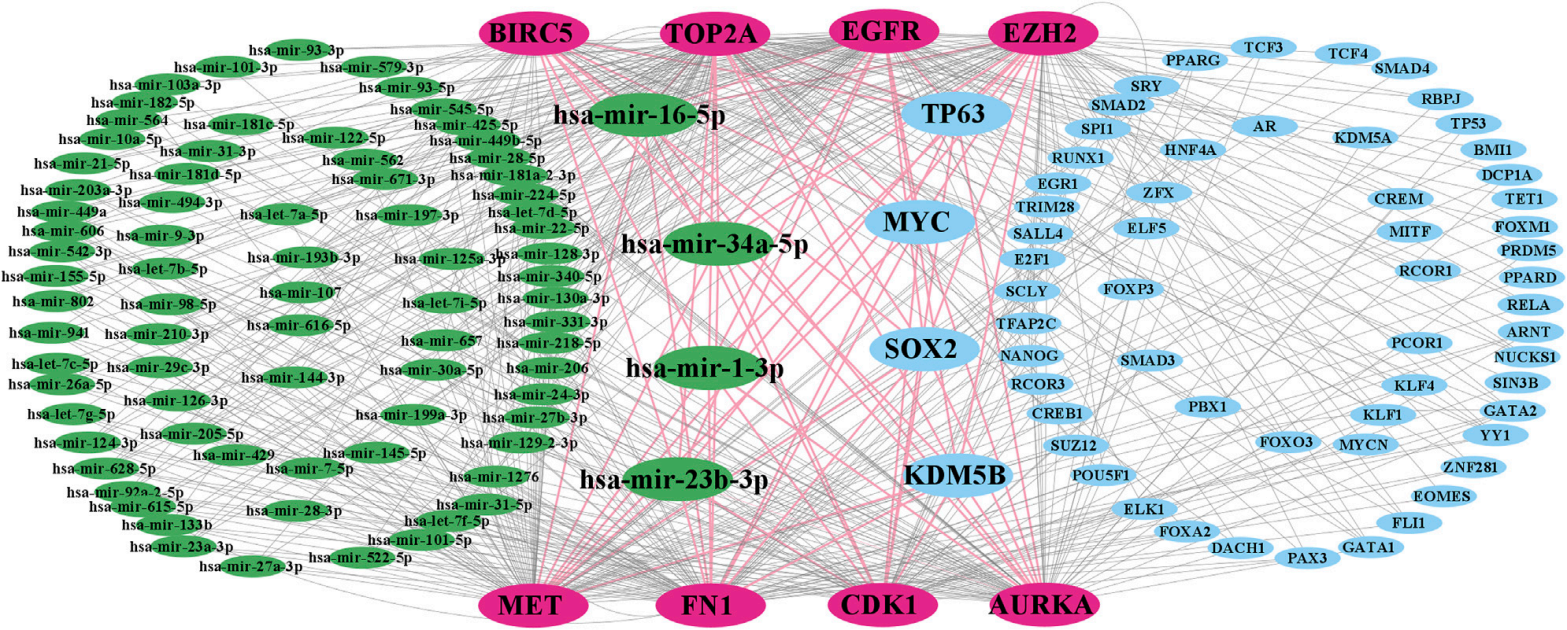
TF-KG–miRNA interaction network, where pink indicates KGs, green indicates miRNAs, blue indicates TFs, and large green and blue indicate key miRNAs and TFs, respectively.

Regulatory interaction networks (TFs-KGs-miRNAs) are visualized in Figure 4, where green indicates miRNAs, pink indicates KGs, blue indicates TFs, and large size indicates key factors. We observed that the four TFs, such as TP63, MYC, SOX2, and KDM5B are associated with all KGs; therefore, we considered these four TFs to be key transcriptional regulators of KGs. Similarly, the four miRNAs, including hsa-mir-16-5p, hsa-mir-34a-5p, hsa-mir-1-3p, and hsa-mir-23b-3p were also associated with all KGs; thus, we considered these four miRNAs to be key posttranscriptional regulators of KGs.

### Validation of the expression pattern of key genes

**FIGURE 5:**
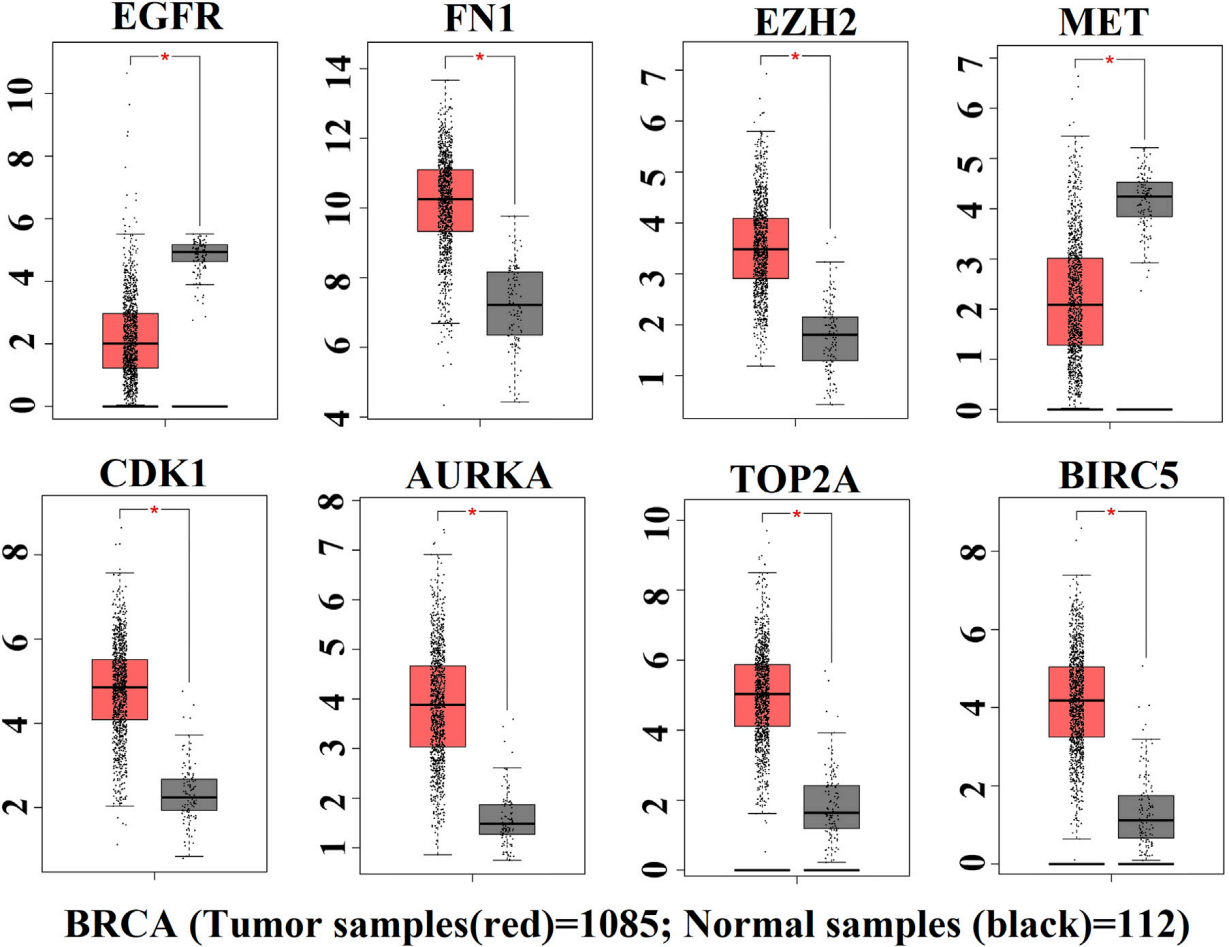
Box plot of expression patterns of KGs for RNA-seq data, where red indicates the high-risk group and black indicates the low-risk group.

The expression patterns of KGs are displayed by box plots for independent data (TCGA RNA-seq data) in Figure 5. We observed that the expression patterns of all KGs were highly differentiated; among them, six KGs (FN1, EZH2, CDK1, AURKA, TOP2A, and BIRC5) were upregulated, and the remaining two KGs (EGFR and MET) were downregulated, which supported our original results.

### Prognostic power analysis of key genes

**FIGURE 6:**
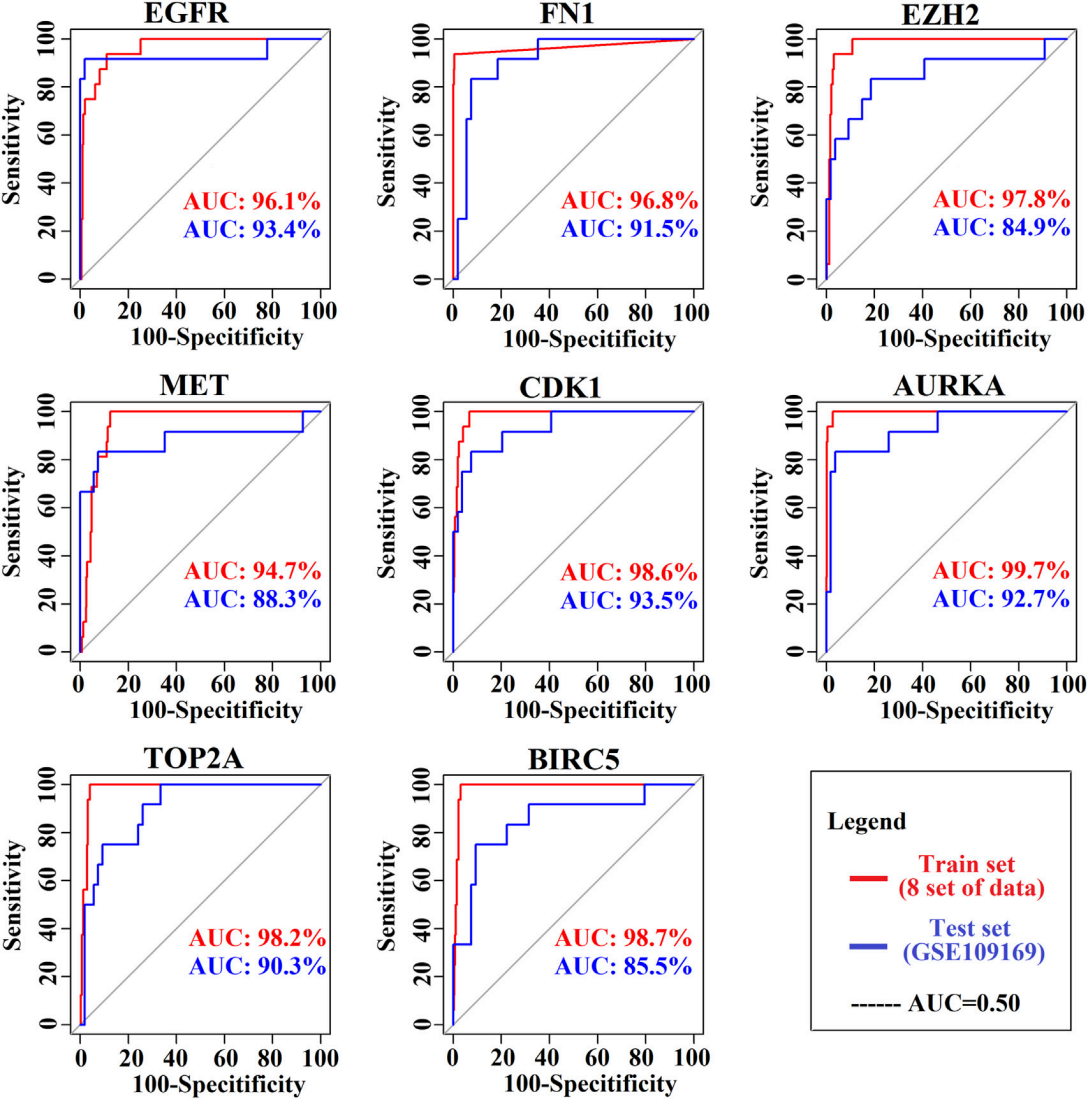
Prognostic powers of KGs were represented by ROC curves, where red indicates the prediction curve and blue indicates the validation curve.

The ROC curves of the cancer prediction/classification model to test the prognostic power of our proposed KGs are presented in Figure 6. The AUC ranged from 94.7 to 99.7 for the prediction set (red color) and 85.5 to 93.5 for the validation set (blue color). Overall, EGFR, FN1, EZH2, MET, CDK1, AURKA, TOP2A, and BIRC5 achieved the best performance (AUC > 85.5) for each of the training and independent test datasets, which indicates that there is a strong prognostic power of the identified KGs for discriminating between the tumor and the normal samples in BC patients.

### Exploring repurposing candidate drugs by molecular docking analysis

**FIGURE 7:**
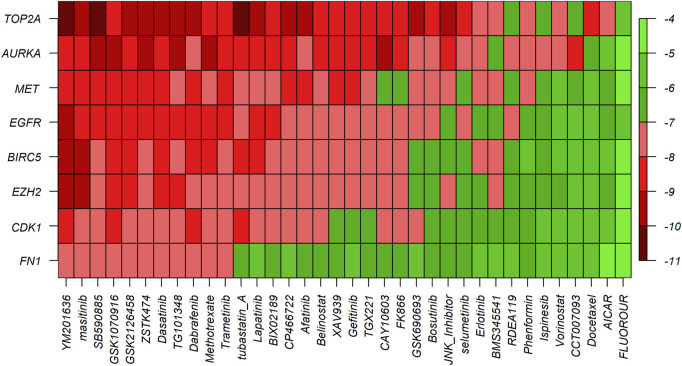
Molecular docking analysis results for exploring candidate drugs against BC. The *Y*-axis presents proteins (targets), the *X*-axis presents compounds (drugs), and different colors indicate the binding affinity score (BAS).

To explore the candidate drugs for BC, we considered eight KG-based proteins EGFR, FN1, EZH2, MET, CDK1, AURKA, TOP2A, and BIRC5 as drug targets. We collected eight KG-associated drugs from the GSCALite database and considered them meta-drug agents. Then, we performed molecular docking analysis between our proposed receptors and the meta-drug agents. The binding affinity score matrix between the ordered receptors and the ordered drug agents is displayed in Figure 7. We observed that the top-order eleven leading compounds/drugs (YM201636, masitinib, SB590885, GSK1070916, GSK2126458, ZSTK474, dasatinib, TG101348, dabrafenib, methotrexate, and trametinib) produced highly significant binding affinity scores (BAS) < −7 with all target proteins. Next, six drugs (tubastatin_A, lapatinib, BIX02189, CP466722, afatinib, and belinostat) had highly significant BAS < −7 with seven target proteins. The drug laptinib was approved by the FDA in 2007 for BC. Therefore, we suggest in this computational study that 16 repurposing candidate drugs may be effective against BC patients (Table 5).

**TABLE 5:** List of 16 proposed repurposing drugs for BC.

Drugs	PubMed CID	Status	Diseases	Targets
YM201636	9956222	*In vivo*	Liver cancer	PIKfyve
		Approval was denied by the EU in 2017 and 2018	Mast cell disease and amyotrophic lateral sclerosis	PDGFR, LCK, FAK, FGFR3, and CSF1R
Masitinib	10074640	Clinical trials	Alzheimer’s disease, malignant melanoma, mastocytosis, multiple myeloma, gastrointestinal cancer, pancreatic cancer, asthma, and COVID-19	
SB590885	135421339	NA	Hepatocellular carcinoma	BRAF
GSK1070916	46885626	Clinical trials (phase 1)	Advanced solid tumors	AURKB, AURKC
GSK2126458	25167777	Clinical trials (phase 1)	Solid tumors	PI3K, MTOR
ZSTK474	11647372	Clinical trials (phase 1)	Neoplasms	PI3K, MTOR
Dasatinib	3062316	Approved by FDA in 2010&2017	Adults with CP-CML & children with Ph + -CML	BCR-ABL, SRC, and C-KIT
		Clinical trials (phase 3)	Myeloid leukemia, chronic	
TG101348/Fedratinib	16722836	Approved by FDA in 2019	Myeloproliferative neoplasms (MPNs)	JAK2, FLT3, and RET
		Clinical trials (phase 3)	Primary myelofibrosis, postpolycythemia vera, and myelofibrosis	
Dabrafenib	44462760	Approved by FDA in 2013	BRAF V600E mutation-positive advanced melanoma	RAF, BRAF, CRAF, MEK
		Clinical trials (phase 3)	Melanoma	
Methotrexate	126941	It was first made in 1947	Cancer	DHFR
		Clinical trials (phase 4)	Rheumatoid Arthritis	
Trametinib	11707110	Approved by FDA in 2013	V600E mutated metastatic melanoma	BRAF, MEK, MEK1 and MEK2
		Clinical trials (phase 1)	Advanced malignant solid neoplasm, metastatic malignant neoplasm in the liver, metastatic malignant solid neoplasm, and unresectable solid neoplasm	
Tubastatin A	49850262	NA	NA	NA
Lapatinib^a^	208908	Approved by FDA in 2007	Breast cancer	EGFR, HER2
BIX02189	135659062	Clinical trials	Kidney Cancer	MEK, ERK
CP466722	44551660	NA	Cancer	ATM, ATR
Afatinib	10184653	Clinical trials (phase 3)	Non-small cell lung cancer	EGFR
Belinostat	6918638	Approved by FDA in 2014	Peripheral T-cell lymphoma	HDAC

a The drug already published/under clinical trials.

### Induction of breast cancer cell death by the candidate drug masitinib

**FIGURE 8:**
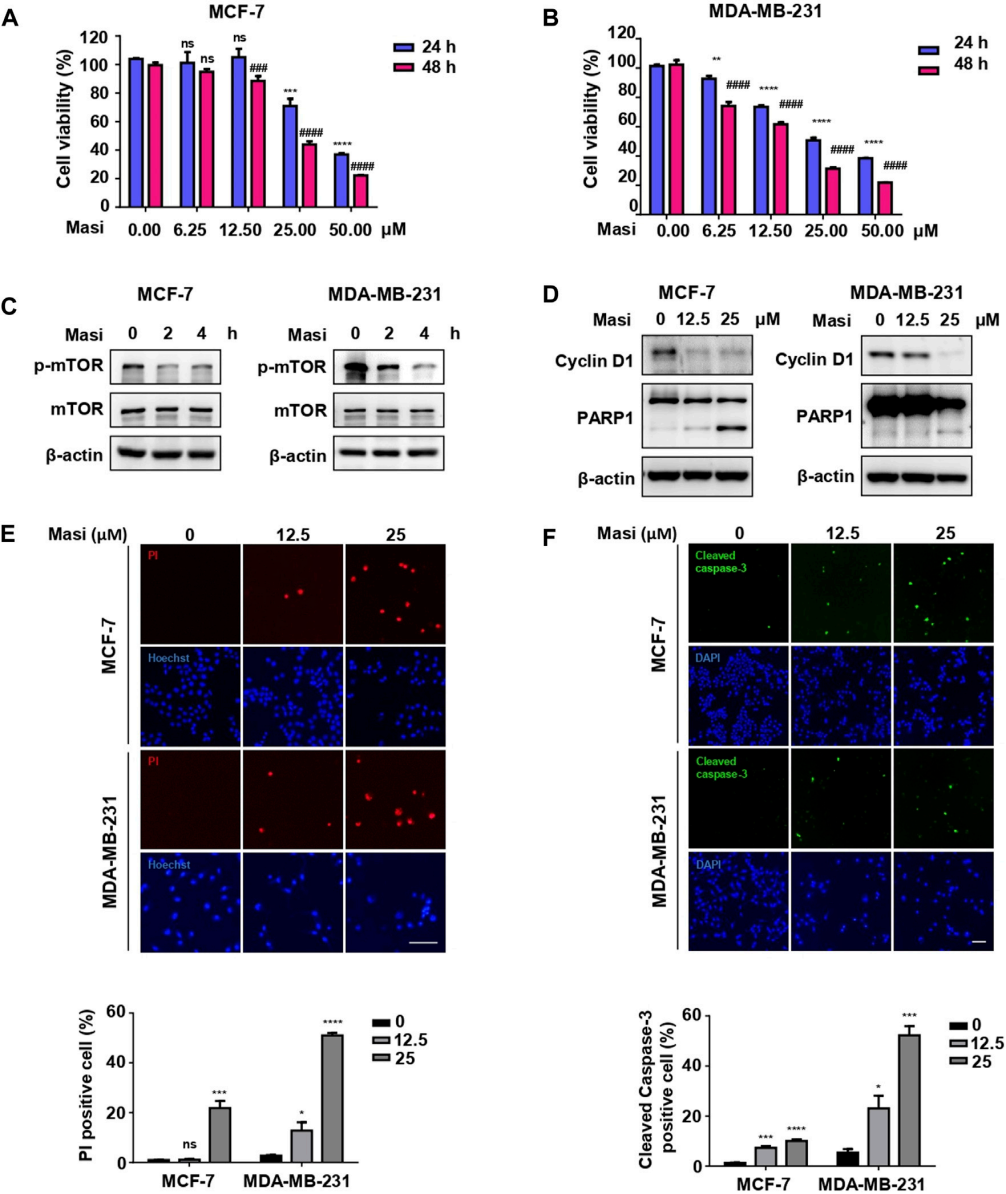
Masitinib treatment effectively killed MCF-7 cells and MDA-MB-231 cells in a dose- and time-dependent manner. **(A)** MCF-7 cells were treated with different dosages of masitinib (0, 6.25, 12.5, 25, and 50 μM) or DMSO for 48 h. Cell viability was determined by CCK-8 assay at different time points (24 and 48 h). The values are presented as the mean ± SEM from three independent experiments. ^***^ *p* < 0.001, ^****^ *p* < 0.0001, ns, no significant difference vs. 24 h DMSO group, one-way ANOVA followed by Dunnett’s multiple-comparisons test. ^###^ *p* < 0.001, ^####^ *p* < 0.0001, ns, no significant difference vs. 48 h DMSO group, one-way ANOVA followed by Dunnett’s multiple-comparisons test. (Masi: masitinib). **(B)** MDA-MB-231 cells were treated with different dosages of masitinib (0, 6.25, 12.5, 25, and 50 μM) or DMSO for 48 h. Cell viability was determined by CCK-8 assay at different time points (24 and 48 h). The values are presented as the mean ± SEM from three independent experiments. ^**^ *p* < 0.01, ^****^ *p* < 0.0001 vs. 24 h DMSO group, one-way ANOVA followed by Dunnett’s multiple-comparisons test. ^####^ *p* < 0.0001 vs. 48 h DMSO group, one-way ANOVA followed by Dunnett’s multiple-comparisons test. (Masi: masitinib). **(C)** Masitinib treatment inhibited the phosphorylation levels of mTOR protein in MCF-7 cells and MDA-MB-231 cells. MCF-7 cells and MDA-MB-231 cells were treated with 25 μM masitinib separately. The protein levels of p-mTOR, mTOR, and β-actin were measured using immunoblot analyses at different time points (0, 2, and 4 h). (Masi: masitinib). **(D)** MCF-7 cells were treated with different dosages of masitinib (0, 12.5, and 25 μM) for 48 h. MDA-MB-231 cells were treated with different dosages of masitinib (0, 12.5, and 25 μM) for 24 h. Then, the protein levels of Cyclin D1, PARP1, and β-actin in these two types of cells were measured using immunoblot analyses. (Masi: masitinib). **(E)** MCF-7 cells and MDA-MB-231 cells were treated as in **(D)** and then incubated with propidium iodide (PI) and Hoechst to detect the cell states. Scale bar, 100 μm. The PI-positive cells were counted and quantified with cell numbers marked with Hoechst. The values are presented as the mean ± SEM from three independent experiments. ^*^ *p* < 0.05, ^***^ *p* < 0.001, ^****^ *p* < 0.0001, ns, no significant difference vs. DMSO group, one-way ANOVA followed by Dunnett’s multiple-comparisons test. **(F)** MCF-7 cells and MDA-MB-231 cells were treated as in **(E)** and then stained with anti-cleaved caspase-3 antibody and DAPI. Scale bar, 100 μm. The cleaved caspased-3-positive cells were counted and quantified with cell numbers marked with DAPI. The values are presented as the mean ± SEM from three independent experiments. ^*^ *p* < 0.05, ^***^ *p* < 0.001, ^****^ *p* < 0.0001 vs. DMSO group, one-way ANOVA followed by Dunnett’s multiple-comparisons test.

To further confirm the potential roles of the candidate drugs on BC, we selected one of the candidates masitinib to examine the effects on two BC cell lines. In both MCF-7 (Figure 8A) and MDA-MB-231 (Figure 8B) cells, masitinib decreased cell viability in a dose-dependent manner. Moreover, masitinib inhibited the phosphorylation of mTOR (Figure 8C). Furthermore, masitinib decreased the levels of cyclin D, an essential regulator of the G1 to S phase transition and increased the cleavage of PARP1 (Figure 8D), suggesting that masitinib influences the cell cycle and induces apoptosis. The induction of apoptotic cell death by masitinib was further confirmed with PI staining (Figure 8E) and immunofluorescent staining using anti-cleaved caspase-3 antibodies (Figure 8F), showing that with higher doses of masitinib, more cells were labeled with PI and cleaved caspase-3 in both MCF-7 and MDA-MB-231 cells.

## Discussion

BC is the cause of a high prevalence and leading mortality rate for women worldwide and is constantly increasing every year. The 5-year overall survival rate for BC patients is still very low, although there has been significant progress in systematic treatment over the past few decades. Therefore, research needs to be improved to discover potential molecular targets and effective candidate drugs that could play a significant role in improving survival rates and reducing mortality in BC patients.

In this study, we identified the potential biomarkers and the candidate drugs by highlighting their pathogenetic processes, transcriptional and posttranscriptional regulatory factors, expression levels, prognostic power, and drug molecules through integrated bioinformatics and network-based techniques. First, we screened 68 oDEGs between BC and normal samples using gene expression profiles. Then, we selected BC-causing two downregulated KGs (EGFR and MET) and six upregulated KGs (FN1, EZH2, CDK1, AURKA, TOP2A, and BIRC5) through the PPI network analysis (Figure 2; Table 3). Some studies have also proposed our identified KGs as BC-causing genes (Navolanic et al., 2003; Gastaldi et al., 2010; Li et al., 2017; Komoto et al., 2018; Deng et al., 2019). In particular, EGFR and its downstream signaling pathways are related to the progression of BC and play an important role against BC treatment using cytotoxic drugs (Navolanic et al., 2003). Analysis of gene expression profiles revealed that MET oncogene immunoreactivity is significantly higher in the progression of basal-like BC in humans than in other types of cancer (Gastaldi et al., 2010). Decreased stability of EZH2 is responsible for the progression and metastasis of BC, but ANCR plays an important role in controlling the stability of EZH2 which inhibits the progression of BC (Li et al., 2017). Bioinformatic analysis has proposed CDK1 as an upregulated gene related to BC progression and tumorigenesis (Deng et al., 2019). Chalcones inhibited the expression of AURKA protein and affected the anti-resistance and anti-metastatic properties of the BC cell lines MCF-7 and BT-20 (Komoto et al., 2018). Based on *in silico* analysis proposed that TOP2A is related to BC tumorigenesis and progression and has been highlighted as a pathogenetic process (Deng et al., 2019). BIRC5 was proposed as an upregulated gene and could be a significant marker for the detection and prognosis of BC at an early age (Ghaffari et al., 2016; Wang et al., 2018).

To investigate the pathogenetic processes of KGs, we selected four BP terms, six CC terms, three MF terms, and five KEGG terms based on directly associated with at least two KGs from the top ten terms of each category (Figure 3). Several studies also claimed that our identified KG-associated functional and pathway terms are responsible for BC progression (Emmert-Streib et al., 2014; Liu B. et al., 2018; Xiu et al., 2019; Han et al., 2020; Hermawan et al., 2020; Liu et al., 2020; Peng et al., 2021; Xiao et al., 2021; Zeng et al., 2021). GO functional analysis revealed that the BC-causing genes were significantly enriched in nuclear division (associated with KGs: TOP2A, AURKA, and BIRC5), organelle fission (associated with KGs: EGFR, AURKA, and BIRC5), and mitotic nuclear division (associated with KGs: MET and BIRC5) (Emmert-Streib et al., 2014; Han et al., 2020; Xiao et al., 2021). The three CC terms are condensed chromosome (associated with KGs: TOP2A, AURKA, and BIRC5), chromosomal region (associated with KGs: CDK1, AURKA, BIRC5, and EZH2) and midbody (associated with KGs: CDK1, AURKA, and BIRC5), which are associated with BC progression (Xiu et al., 2019; Liu et al., 2020). The MF term protein serine/threonine kinase activity (associated with KGs: CDK1, AURKA, and EGFR) is responsible for BC progression (Hermawan et al., 2020). Finally, the four KEGG pathway terms focal adhesion (associated with KGs: FN1, EGFR, and MET), progesterone-mediated oocyte maturation (associated with KGs: CDK1 and AURKA), oocyte meiosis (associated with KGs: CDK1 and AURKA), and adherens junction (associated with KGs: EGFR and MET), were also related to BC progression (Liu B. et al., 2018; Han et al., 2020; Peng et al., 2021; Zeng et al., 2021).

Four TFs (TP63, MYC, SOX2, and KDM5B) and four miRNAs (hsa-mir-16-5p, hsa-mir-34a-5p, hsa-mir-1-3p, and hsa-mir-23b-3p) were considered transcriptional and posttranscriptional and were connected to all KGs in the regulatory interaction network (Figure 4). Furthermore, we also checked the prognostic power of KGs for BC patients through the popular machine learning algorithm SVM. We developed a prediction model through SVM and showed good performance for both the training (GSE54002, GSE29431, GSE124646, GSE42568, GSE45827, GSE10810, GSE65216, and GSE36295) and test (GSE109169) (independent data) datasets. We found AUC values for eight KGs through the training dataset: EGFR (AUC: 96.1), FN1 (AUC: 96.8), EZH2 (AUC: 97.8), MET (AUC: 94.7), CDK1 (AUC: 98.6), AURKA (AUC: 99.7), TOP2A (AUC: 98.2), and BIRC5 (AUC: 98.7). Similarly, we found AUC values for eight KGs through the test dataset: EGFR (AUC: 93.4), FN1 (AUC: 91.5), EZH2 (AUC: 84.9), MET (AUC: 88.3), CDK1 (AUC: 93.5), AURKA (AUC: 92.7), TOP2A (AUC: 90.3), and BIRC5 (AUC: 85.5) (Figure 6). We noticed that the predictor model showed good performance with independent test datasets. These results indicate that our identified KGs have strong prognostic power for BC patients.

To discover potential candidate repurposing drugs for the treatment against BC patients, 77 KG-related drug agents were selected from the GSCALite database. Among them 16 candidate drugs (YM201636, masitinib, SB590885, GSK1070916, GSK2126458, ZSTK474, dasatinib, fedratinib, dabrafenib, methotrexate, trametinib, tubastatin A, BIX02189, CP466722, afatinib, and belinostat) were proposed based on BAS < −7 through molecular docking analysis (Figure 7; Table 5). Some of our proposed 16 drugs have already been approved, some are under clinical trial, and some are under *in silico, in vivo,* and *in vitro* analyses (Folch et al., 2015; Lee et al., 2015; Agency, 2017; FDA, 2017; European Commission, 2018; Odogwu et al., 2018; FDA, 2019; Hou et al., 2019; Drayman et al., 2021). Specifically, the drug YM201636 has been recommended which is effective against the progression of liver cancer through PIKfyve target inhibition (Hou et al., 2019). The drug masitinib was approved for mast cell disease and amyotrophic lateral sclerosis but later denied approval by the EU in 2017 and 2018 (Agency, 2017; European Commission, 2018). This drug has been studied for various human diseases and is now under clinical trial such as COVID-19 and Alzheimer’s disease (Folch et al., 2015; Drayman et al., 2021). Dasatinib was approved by the FDA in 2010 and 2017 for the treatment of adults with CP-CML and children with Ph + -CML, respectively (FDA, 2017). Fedratinib was approved by the FDA on 16 August 2019 for the treatment of MPN disease (FDA, 2019). Dabrafenib was approved by the FDA on 29 May 2013 for the treatment of BRAF V600E mutation-positive advanced melanoma disease (Odogwu et al., 2018). Tramatinib was approved by the FDA in May 2013 for the treatment of V600E mutated metastatic melanoma disease (Odogwu et al., 2018). Belinostat was approved by the FDA in 2013 for the treatment of peripheral T-cell lymphoma disease (Lee et al., 2015). However, the current study emphasizes experimental-lab verification for proposed candidate drugs with target proteins for treatment against BC.

Masitinib is an antitumor drug used primarily for mast cell tumors, but also used in solid tumors (Hahn et al., 2008). It inhibits the tyrosine kinase c-kit of mast cells, leading to a decrease in cell proliferation (Hahn et al., 2008). In BC, the cell surface receptors, including EGFR, HER-2, c-MET, and Trop2A, are cell surface markers that are potential targets for drugs (Butti et al., 2018). EGFR activation can induce the PI3K/AKT/mTOR signaling pathway to promote cell growth and proliferation (Miyamoto et al., 2017). Hyperactivation of the PI3K/AKT/mTOR pathway is commonly observed in BC, including triple-negative BC (Hussain et al., 2022). Inhibition of PI3K/AKT/mTOR by a dual PI3K/mTOR inhibitor apitolisib induces apoptotic cell death and decreases cell proliferation (Omeljaniuk et al., 2021). In the present study, we identified that masitinib as a candidate drug that potentially targets multiple BC-related products, including the cell surface marker EGFR. Using the breast cancer cell lines MCF-7 and MDA-MB-231, we observed that masitinib inhibits the activity of mTOR, a downstream effector of EGFR. It is well-documented that mTOR signaling is involved in cell proliferation, growth, metabolism, and survival (Zoungrana et al., 2022). In our observations, masitinib decreased cell migration and induced apoptotic cell death in MCF-7 and MDA-MB-231 cells. Thus, our data suggest that masitinib possibly acts on the EGFR/mTOR pathway to mediate antitumor activity.

## Conclusion

In this study, we identified eight stable BC-guided KGs EGFR, FN1, EZH2, MET, CDK1, AURKA, TOP2A, and BIRC5 using well-established bioinformatics and network-based tools and highlighted their pathogenetic processes, regulatory factors, prognostic power, and drug molecules. Then, we proposed 16 potential candidate repurposing drugs YM201636, masitinib, SB590885, GSK1070916, GSK2126458, ZSTK474, dasatinib, fedratinib, dabrafenib, methotrexate, trametinib, tubastatin A, BIX02189, CP466722, afatinib, and belinostat through molecular docking analysis. Furthermore, we verified the proposed results through literature review and different databases. Finally, we examined one of the candidates masitinib to identify the anti-breast cancer effects. We found that masitinib inhibits the mTOR signaling pathway and induces apoptotic cell death. Therefore, the proposed molecular biomarkers and repurposing candidate drugs may play an important role in the diagnosis and therapy of BC.

## Data Availability

Publicly available datasets were analyzed in this study. This data can be found here: https://www.ncbi.nlm.nih.gov/geo/query/acc.cgi?acc=GSE54002 https://www.ncbi.nlm.nih.gov/geo/query/acc.cgi?acc=GSE29431 https://www.ncbi.nlm.nih.gov/geo/query/acc.cgi?acc=GSE124646 https://www.ncbi.nlm.nih.gov/geo/query/acc.cgi?acc=GSE42568 https://www.ncbi.nlm.nih.gov/geo/query/acc.cgi?acc=GSE45827 https://www.ncbi.nlm.nih.gov/geo/query/acc.cgi?acc=GSE10810 https://www.ncbi.nlm.nih.gov/geo/query/acc.cgi?acc=GSE65216 https://www.ncbi.nlm.nih.gov/geo/query/acc.cgi?acc=GSE36295 https://www.ncbi.nlm.nih.gov/geo/query/acc.cgi?acc=GSE109169.
