# Supplementary material for: Bioinformatics and network-based screening and discovery of potential molecular targets and small molecular drugs for breast cancer
Source: Front Pharmacol. 2022 Sep 20;13:942126. doi: 10.3389/fphar.2022.942126 (PMC9531711; doi:10.3389/fphar.2022.942126)
Supplement: Supplementary file 1 [file DataSheet1.PDF]

Supplementary material

Bioinformatics and Network-Based Screening and Discovery of Potential Molecular Targets and Small Molecular Drugs for Breast Cancer

Md Shahin Alam[1#], Adiba Sultana[1#], Hongyang Sun[1#], Jin Wu[1],Fanfan Guo[2], Qing Li[3], Haigang Ren[1], Zongbin Hao[1*], Yi Zhang[2*], and Guanghui Wang[1*]

[1]Laboratory of Molecular Neuropathology, Department of Pharmacology, Jiangsu Key Laboratory of Neuropsychiatric Diseases and College of Pharmaceutical Sciences, Soochow University, 199 Ren'ai Road, Suzhou 215123, Jiangsu, China.

[2]Department of Pharmacology, College of Pharmaceutical Science, Soochow University, Suzhou, China.

[3]Department of Gastroenterology, the First People's Hospital of Taicang, Taicang Affiliated Hospital of Soochow University, Suzhou, Jiangsu 215400, China

[#]These authors contributed equally to this study

*Correspondence: Zongbin Hao, Guanghui Wang or Yi Zhang (haozb835@126.com, or zhangyi@suda.edu.cn, or wanggh@suda.edu.cn)

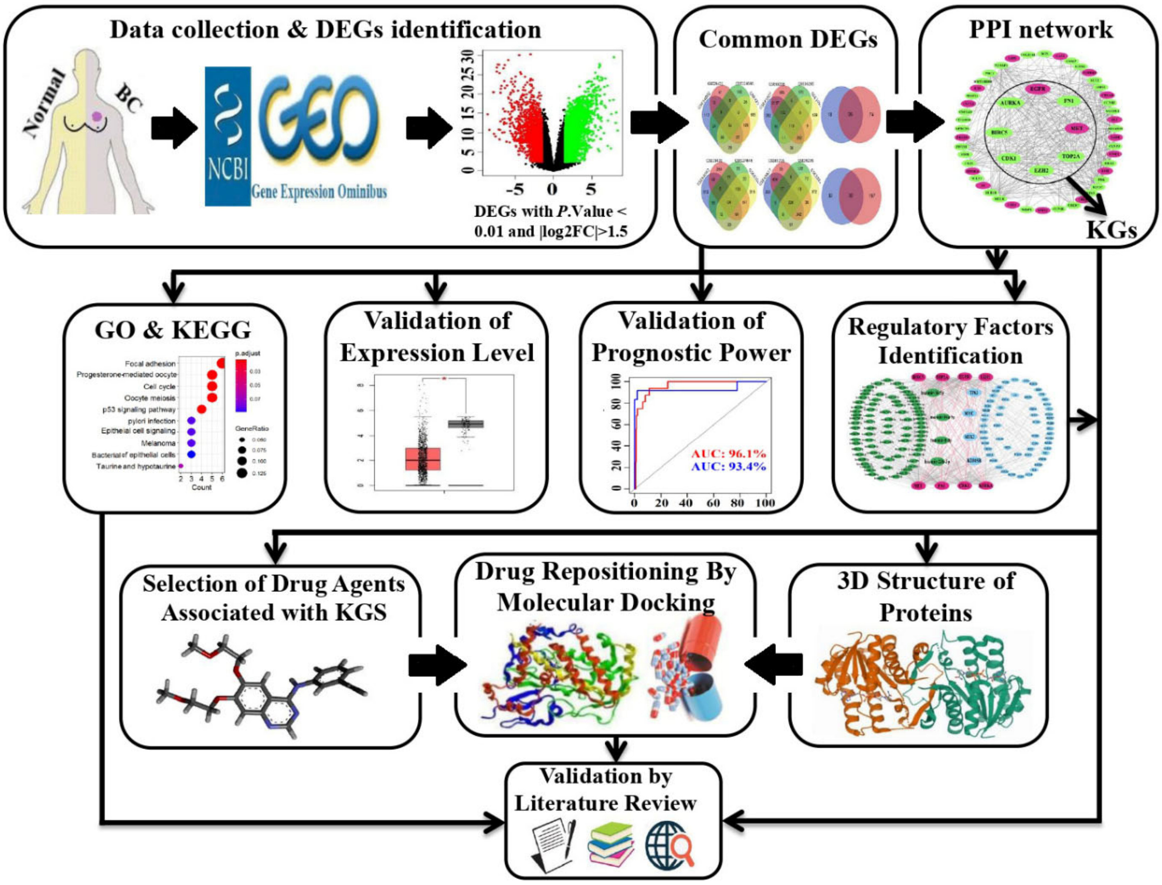

FIGURE S1. The pipeline of the current study. BC: breast cancer; oDEGs: overlapping differentially expressed genes; PPI: protein–protein interaction; KGs: key genes; GO: Gene Ontology; KEGG: Kyoto Encyclopedia of Genes and Genomes; 3D: 3 dimensional.
